# Cardiovascular safety of febuxostat compared to allopurinol for the treatment of gout: A systematic and meta‐analysis

**DOI:** 10.1002/clc.23643

**Published:** 2021-05-20

**Authors:** Linggen Gao, Bin Wang, Ying Pan, Yan Lu, Rui Cheng

**Affiliations:** ^1^ Department of Comprehensive Surgery General Hospital of Chinese People's Liberation Army & National Clinical Research Center for Geriatric Disease Beijing China

**Keywords:** allopurinol, cardiovascular safety, febuxostat

## Abstract

The cardiovascular safety of febuxostat compared to allopurinol for the treatment of gout remains equivocal. Febuxostat had a better safety outcome compared with allopurinol. In this systematic review and meta‐analysis, we searched MEDLINE and Embase for articles published between March 1, 2000 and April 4, 2021, without any language restrictions. We did a systematic review and meta‐analysis of included clinical trials to evaluate the cardiovascular safety of febuxostat compared to allopurinol for treatment of chronic gout. Two reviewers independently selected studies, assessed study quality, and extracted data. Risk ratios were calculated with random effects and were reported with corresponding 95% confidence intervals (CI). From 240 potentially relevant citations, 224 papers were excluded; 16 studies were ultimately included in the analysis. Febuxostat had a better safety outcome compared with allopurinol，which was the composite of urgent coronary revascularization (OR: 0.84, 95% CI: 0.77–0.90, *p* < .0001) and stroke (OR: 0.87, 95% CI: 0.79–0.97, *p* = .009). However, that difference was not found in nonfatal myocardial infarction (OR: 0.99, 95% CI: 0.80–1.22, *p* = .91), cardiovascular related mortality (OR: 0.98, 95% CI: 0.69–1.38, *p* = .89) and all‐cause mortality (OR: 0.93, 95% CI: 0.75–1.15, *p* = .52). No significant differences in cardiovascular related mortality and all‐cause mortality were observed across any subgroup. This meta‐analysis adds new evidence regarding the cardiovascular safety of febuxostat in patients. Initiation of febuxostat in patients was not associated with an increased risk of death or serious cardiovascular related adverse events compared with allopurinol.

## 1 INTRODUCTION

Gout is a common clinical metabolic system disease and may contribute to many adverse health events. Evidence shows that the risk of hyperuricemia increased with advanced age in both sexes.^1^, ^2^ At present, drugs are the first choice for the treatment of gout in clinical practice. Studies have found that the treatment of gout with xanthine oxidase inhibition (allopurinol, febuxostat) can increase uric acid excretion via the kidneys and achieve better results. Recent studies have shown that febuxostat, a novel non‐purine selective inhibitor of xanthine oxidase (XO), is more effective than allopurinol in lowering the uric acid levels in patients with hyperuricemia and gout.^3^, ^4^ It is particularly useful in patients who are refractory or intolerant to allopurinol, and requires no dose limitation in stages 1–3 chronic kidney disease.^5^ However, the food and drug administration (FDA) issued a public safety alert, responding to results of cardiovascular safety of febuxostat and allopurinol in patients with gout and cardiovascular morbidities (CARES) trial.^6^ The FDA public safety alert highlights the discussion of CV safety of febuxostat.^7^, ^8^, ^9^ By contrast, the European Medicines Agency (EMA)‐required febuxostat versus allopurinol streamlined trial, a prospective, randomized, open‐label, blinded‐endpoint, non‐inferiority trial of febuxostat (80–120 mg/day) versus allopurinol, does not support the finding of an increased cardiovascular risk of febuxostat.^10^ The evidence for a causal relationship between xanthine oxidase inhibitors and cardiovascular diseases (CVD) remains equivocal. Therefore, this study intends to conduct a systematic review of the relevant clinical trials published in recent years to analyze the adverse cardiovascular events and death risks of febuxostat compared with allopurinol in patients.

## 2 METHODS

### 2.1 Search strategy and selection criteria

We followed the Preferred Reporting Items for Systematic Reviews and Meta‐Analyses (PRISMA) guidelines in this systematic review and meta‐analysis.^11^ We systematically searched clinical trials of febuxostat and allopurinol treatment of gout in the elderly in PubMed, EMBASE, the Cochrane Library database and reviews of relevant articles from January 2000 to April 4, 2021. The following terms were used: “Gout” “Febuxostat” “Allopurinol” OR “Clinical Trial” “adverse events.” Language of publication did not influence article selection. Titles and abstracts were screened to exclude ineligible studies.

Studies were included if they met the following criteria: (i) clinical trials; (ii) treatment status as treated with febuxostat and allopurinol;(iii) long‐term follow up of patients.

Exclusion criteria: (i) Documents in languages other than Chinese and English. (ii) There are no statistics on cardiovascular and death‐related adverse events for the outcome indicators, and the data is incomplete. (iii) Patients with severe liver and kidney dysfunction, unstable vital signs, long‐term alcoholism, and other conditions that will affect the resolution of indicators (iv) Patients with secondary gout.

Gao LG and Bin Wang screened titles, abstracts, and full text of papers identified in our search and assessed for risk of bias.

The titles of the primary 240 publications identified were reviewed and 224 were discarded although they were identified by our search terms. The studies were also discarded because the enrolled patients with acute hyperuricemia or secondary hyperuricemia (e.g., end‐stage renal disease). Finally, 14 publications were chosen for the meta‐analysis.

### 2.2 Study groups and clinical evaluation

The study population in the present meta‐analysis consisted of 257 851 patients. Patients were categorized by treatment status as treated with febuxostat or allopurinol. The details in the pharmacologic intervention were listed in Table 1. All patients underwent complete clinical evaluations and fulfilled the diagnostic criteria. Outcomes of major events from each trial were selected, which were consisted of cardiovascular related mortality, major vascular events (including myocardial infarction or other acute coronary syndrome, coronary revascularization, or stroke, etc.) and all‐cause mortality.

**TABLE 1:** Baseline characteristics of including studies

Study	Comparison	Year of publication	Study design	No. of patients	Median age	Population	Male sex — no. (%)	Hypertension	Hyperlipidemia
Ju et al.	Febuxosta	2020	Retrospective cohort study	276	70.41 (14.35)	Chinese	186 (67.4)	‐	123 (44.6)
Ju et al.	Allopurinol	2020	Retrospective cohort study	828	70.01 (14.90)	Chinese	549 (66.3)	‐	374 (45.2)
Becker et al.	Febuxostat (80 mg)	2005	P; R; O	256	51.8 ± 11.7	White Race75%	243 (95)	106 (41)	90 (35)
Becker et al.	Febuxostat (120 mg)	2005	P; R; O	251	52.0 ± 12.1	White Race 79%	243 (97)	113 (45)	79 (31)
Becker et al.	Allopurinol (300 mg)	2005	P; R; O	253	51.6 ± 12.6	White Race 77%	243 (96)	112 (44)	86 (34)
Becker et al.	Febuxostat (80 mg)	2009	P; R; O	649	51.4 ± 11.95	White Race 80%	>90%^b^	295 (45.5)	229 (35.3)
Becker et al.	Febuxostat (120 mg)	2009	P; R; O	292	50.9 ± 11.57	White Race 79.8%	>90%^b^	115 (39.4)	89 (30.5)
Becker et al.	Allopurinol (300 mg)	2009	P; R; O	145	51.0 ± 11.30	White Race 75.9%	>90%^b^	73 (50.3)	47 (32.4)
Becker et al.	Febuxostat (40 mg)	2010	Double‐blind RCT	757	52.5 ± 11.68	White Race (81.9%)	722 (95.4)	‐	299 (39.5)
Becker et al.	Febuxostat (80 mg)	2010	Double‐blind RCT	756	53.0 ± 11.79	White Race (81.7%)	710 (93.9)	‐	308 (40.7)
Becker et al.	Allopurinol (200/300 mg)	2010	Double‐blind RCT	756	52.9 ± 11.73	White Race (82.7%)	709 (93.8)	‐	335 (44.3)
Kamatani et al.	Febuxostat (40 mg)	2011	P; R; O	122	51.6 ± 13.1	Japanese	118 (96.7)	49 (40.2)	51 (41.8)
Kamatani et al.	Allopurinol (200 mg)	2011	P; R; O	121	52.6 ± 14	Japanese	119 (98.3)	32 (26.4)	44 (36.4)
Huang et al.	Febuxostat (40 mg)	2014	Double‐blind RCT	172	46.42 ± 10.90	Chinese	167 (97.1)	54 (31.40)	6 (3.49)
Huang et al.	Febuxostat (80 mg)	2014		172	47.40 ± 11.18	Chinese	169 (98.2)	45 (26.16)	5 (2.91)
Huang et al.	Allopurinol (300 mg)	2014		172	46.17 ± 11.56	Chinese	168 (97.7)	44 (25.58)	2 (1.16)
Xu et al.	Febuxostat (80 mg)	2015	Double‐blind RCT	168	48.2 ± 12.0	Chinese	146 (92.4)	32 (20.3)	13 (8.2)
Xu et al.	Febuxostat (40 mg)	2015	Double‐blind RCT	168	45.5 ± 11.9	Chinese	158 (98.8)	20 (12.5)	15 (9.4)
Xu et al.	Allopurinol (300 mg)	2015	Double‐blind RCT	168	46.6 ± 10.7	Chinese	149 (93.7)	22 (13.8)	11 (6.9)
Tanaka et al.	Febuxostat (40 mg)	2015	P; R; O	21	70.1 ± 9.5	Japanese	19 (90.5)	11 (52)	‐
Tanaka et al.	Allopurinol (300 mg)	2015	P; R; O	19	66.1 ± 7.0	Japanese	16 (84.2)	6 (32)	‐
Nakagomi et al.	Febuxostat	2015	P; R; O	31	69.3 ± 10.0	Japanese	22 (71)	27 (87.1)	30 (96.8)
Nakagomi et al.	Allopurinol	2015	P; R; O	30	71.8 ± 8.0	Japanese	18 (69)	30 (100)	29 (96.7)
Yu et al.	Febuxostat (80 mg)	2016	P; R; O	54	46.0 ± 11.0	Taiwan	53 (98.1)	‐	‐
Yu et al.	Allopurinol (300 mg)	2016	P; R; O	55	45.2 ± 12.0	Taiwan	53 (96.4)	‐	‐
White et al.	Febuxostat	2018	Double‐blind RCT	3098	64.0 (58–71)	White race (69.7%)	2604 (84.1)	2864 (92.4)	2678 (86.4)
White et al.	Allopurinol	2018	Double‐blind RCT	3092	65.0 (58–71)	White race (69.2%)	2592 (83.8)	2851 (92.2)	2702 (87.4)
Su et al.	Febuxostat	2019	Cohort study	44 111	65.0 + 15.7	Taiwan	32 694 (74.1)	30 433 (69.0)	16 566 (37.6)
Su et al.	Allopurinol	2019	Cohort study	44 111	64.1 + 15.0	Taiwan	32 863 (74.5)	30 332 (68.8)	16 497 (37.4)
Kang	Febuxostat	2019	Cohort study	9910	59.4 (12.9)	Korean	78.3	55.4	45.5
Kang	Allopurinol	2019	Cohort study	39 640	59.1 (12.5)	Korean	78.9	54.0	44.8
Kojima et al.	Febuxostat	2019	P; R; O	537	75.4 ± 6.7	Japanese	371 (69)	506 (94.2)	317 (59.0)
Kojima et al.	Allopurinol^c^	2019	P; R; O	533	76.0 ± 6.5	Japanese	368 (69)	501 (94.0)	305 (57.2)
Cicero et al.	Febuxostat	2019	Observational trial	120	75.9 ± 8.9	Italy	79 (65.8)	114 (95.0)	78 (65.0)
Cicero et al.	Allopurinol	2019		135	78.1 ± 6.3	Italy	81 (60)	122 (90.4)	88 (65.2)
Mackenzie et al.	Febuxostat	2020	P; R; O	3063	71.0 (6.4)	99.1% White race	2619 (85·5%)	2345 (76·6%)	‐
Mackenzie et al.	Allopurinol	2020	P; R; O	3065	70.9 (6.5)	99.1% White race	2606 (85.0%)	2439 (79.6%)	‐
Zhang et al.	Febuxostat	2020	Cohort study	24 936	76 (70–82)	76.4% White race	52.3	95.4	82.8
Zhang et al.	Allopurinol	2020	Cohort study	74 808	76 (71–82)	76.2% White race	52.3	95.4	82.9

Study	Coronary heart disease	Diabetes (%)	BMI	Follow‐up period	Urgent Coronary revascularisation *n* (%)	Nonfatal myocardial infarction N OR CAD (%)	Cardiovascular death	Nonfatal stroke OR Cerebrovascular disease	Death from any cause
Ju et al.	‐	59 (21.4)	‐	‐	‐	4	0	6	52
Ju et al.	‐	185 (22.3)	‐	‐	‐	19	0	25	204
Becker et al.	23 (9)	17 (7)	32.7 ± 6.1	52 weeks	0	0	1	0	2
Becker et al.	28 (11)	17 (7)	32.3 ± 5.7	52 weeks	0	0	1	0	2
Becker et al.	23 (9)	19 (8)	32.6 ± 6.1	52 weeks	0	‐	0^a^	0^a^	0^a^
Becker et al.	71 (10.9)	46 (7.1)	32.3 ± 5.78	172 weeks	‐	‐	6	‐	7
Becker et al.	33 (11.3)	15 (5.1)	33.2 ± 6.17	172 weeks	‐	‐	‐	‐	3
Becker et al.	14 (9.7)	12 (8.3)	33.8 ± 6.79	172 weeks	‐	‐	0	‐	0
Becker et al.	421 (55.6)	89 (11.8)	‐	28 weeks	‐	0^a^	0^a^	0^a^	1
Becker et al.	440 (58.2)	113 (14.9)	‐	28 weeks	‐	1	0	2	1
Becker et al.	436 (57.7)	110 (14.6)	‐	28 weeks	‐	1	2	0	3
Kamatani et al.		12 (9.8)	‐	8 weeks	0^a^	0^a^	0^a^	0^a^	0^a^
Kamatani et al.		12 (9.9)	‐	8 weeks	0^a^	0^a^	0^a^	0^a^	0^a^
Huang et al.	57 (33.14)	14 (8.14)	25.63 ± 2.80	28 weeks	0	0^a^	0^a^	0^a^	0^a^
Huang et al.	47 (27.33)	9 (5.23)	25.25 ± 2.64	28 weeks	0	0^a^	0^a^	0^a^	0^a^
Huang et al.	45 (26.16)	10 (5.81)	25.44 ± 2.53	28 weeks	0	0^a^	0^a^	0^a^	0^a^
Xu et al.	2 (1.3)	5 (3.2)	25.1 ± 2.6	24 weeks	0	0	0	0	0
Xu et al.	4 (2.5)	10 (6.3)	25.3 ± 2.7	24 weeks	0	0^a^	0^a^	0^a^	0^a^
Xu et al.	4 (2.5)	9 (5.7)	25.4 ± 3.3	24 weeks	0	0^a^	0^a^	0^a^	0^a^
Tanaka et al.	‐	‐	24.1 ± 3.8	12 weeks	0	0	0	0	0
Tanaka et al.	‐	‐	26.1 ± 2.9	12 weeks	0	0	0	0	0
Nakagomi et al.	‐	9 (29.0)	23.6 ± 2.4	23 (13–47)	‐	‐	2 (2/31)	‐	‐
Nakagomi et al.	‐	12 (40.0)	23，1 ± 3.1	23 (13–47)	‐	‐	5 (1/6)	‐	‐
Yu et al.	‐	‐	26.8 ± 3.7	12 weeks	0	0	0	‐	0
Yu et al.	‐	‐	27.8 ± 4.2	12 weeks	0	0	0	‐	0
White et al.	1197 (38.6)	1193 (38.5)	33.6 ± 7.0	72 months	49 (1.6)	111 (3.6)	134 (4.3)	71 (2.3)	243 (7.8)
White et al.	1231 (39.8)	1213 (39.2)	33.4 ± 6.9	72 months	56 (1.8)	118 (3.8)	100 (3.2)	70 (2.3)	199 (6.4)
Su et al.	9390 (21.3)	16 875 (38.3)	‐	28.5 weeks	‐	272 (0.6)	468 (1.06)	344 (0.8)	1630 (3.7)
Su et al.	8582 (19.5)	15 480 (35.1)	‐	22.5 weeks	‐	193 (0.4)	334 (0.75)	298 (0.7)	1301 (2.9)
Kang	1.6	29.5	‐	43.2 weeks	44 (0.4)	20	‐	78	135
Kang	1.6	28.6	‐	35.9 weeks	267 (0.64)	88	‐	382	545
Kojima et al.	45 (8.4)	197 (36.7)	24.74 ± 3.71	152 weeks	2	4	‐	9	10
Kojima et al.	45 (8.4)	199 (37.3)	24.61 ± 3.65	150 weeks	3	7	‐	7	12
Cicero et al.	67 (55.8)	32 (26.7)	25.9 ± 2.9	6 years	‐	‐	8	‐	‐
Cicero et al.	84 (62.2)	39 (28.9)	26.1 ± 1.3	6 years	‐	‐	20	‐	‐
Mackenzie et al.	308 (10.1%)	661 (21·6%)	31.0 (5.1)	72 months	65 (2.1)	102 (3.3%)	62 (2.0)	58 (1.9)	108 (3.5)
Mackenzie et al.	348 (11.4%)	719 (23·5%)	31.2 (5.3)	72 months	78 (2.5)	110 (3·6%)	82 (2·7%)	80 (2.6)	174 (5.7%)
Zhang et al.	3.5	55.0	‐	60	719 (2.8)	457 (2.1)	‐	291 (1.3)	1144 (4.5)
Zhang et al.	3.6	55.2	‐	60	2525 (3.4)	1579 (2.4)	‐	977 (1.5)	4022 (5.3)

Abbreviations: P, prospective; R, randomized; O, open‐label.

^a^ The number of the mortality was not reported directly in these studies but none of the serious adverse events was reported.

^b^ Majority of subjects were male.

^c^ Administration of 100 mg of oral allopurinol was considered if serum uric acid was elevated during the study period.

### 2.3 Data extraction

Two authors (L.G. and B.W.) independently assessed and abstracted relevant trials that met the standardized, predefined criteria. Disagreements were identified computationally. Each was checked independently. If data could not be extracted or calculated from the article with confidence, no data were entered. Any discrepancies between the two reviewers were resolved through discussion. A data extraction form was used to collect the following information: (i) authors, study location, dates of study; (ii) number and age of participants; (iii) study design; (iv) comorbidities; (v) details of administration; (vi) follow‐up time; (vii) outcomes. L.G. and B.W. extracted the data for patients using a standardized data form.

### 2.4 Statistical analysis

The heterogeneity of the included studies was examined by Cochran chisquare tests (*p* < .1). The *I* ^2^ statistic was also examined, and we considered *I* ^2^ > 50% to indicate significant heterogeneity between the trials.^12^ Publication bias was evaluated using both the Begg's funnel plot and the Egger plot. The Mantel–Haenszel^13^ fixed‐effect model or the random‐effects model was chosen for meta‐analysis of the comparison of efficacy and cardiovascular safety of and endpoint events between febuxostat ‐treated group and allopurinol ‐treated group. Statistical analyses were carried out with Review Manager 5.0. *p* values that were <.05 were considered statistically significant. All statistical tests were two‐sided.

To examine the cardiovascular safety and identify the possible source of heterogeneity within these studies, previously defined subgroup analyses were performed (age, population and study design).

## 3 RESULTS

### 3.1 Results of the literature search

**FIGURE 1:**
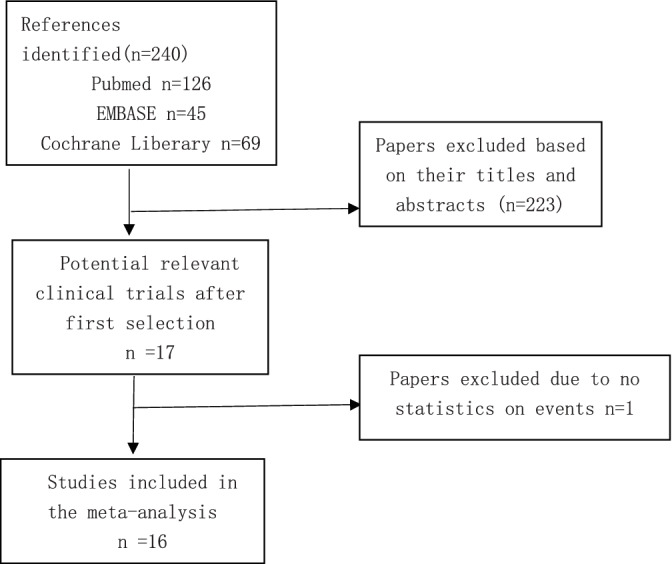
Flow diagram of the study selection procedure, showing the number of citations retrieved by individual searches and the number of trials included in meta‐analysis

Initially, 240 articles were identified from the databases PubMed, EMBASE, and the Cochrane Library. Based on the predefined selection criteria, 224 papers were excluded for different reasons (Figure 1). As a result, 16 clinical trials with 257 851 subjects met all the inclusion criteria and were included in the meta‐analysis (Figure 1).^4^, ^6^, ^10^, ^14^, ^15^, ^16^, ^17^, ^18^, ^19^, ^20^, ^21^, ^22^, ^23^, ^24^, ^25^, ^26^ Demographic data for the patients was shown in Table 1. Among febuxostat users, the median age was from 45.5 to 76.0 years and 52.3%–98.8% were male in the included studies. Among allopurinol users, the median age was from 65.0 to 76.0 years and 52.3%–98.3% were male. In both groups, 1.3%–58.2% had history of coronary heart disease at baseline. Hypertension (12.5%–100%), hyperlipidemia (2.91%–96.8%), and diabetes (3.2%–55.2%) were common comorbidities in both groups.

### 3.2 Effect of febuxostat versus allopurinol treatment on clinical events

**FIGURE 2:**
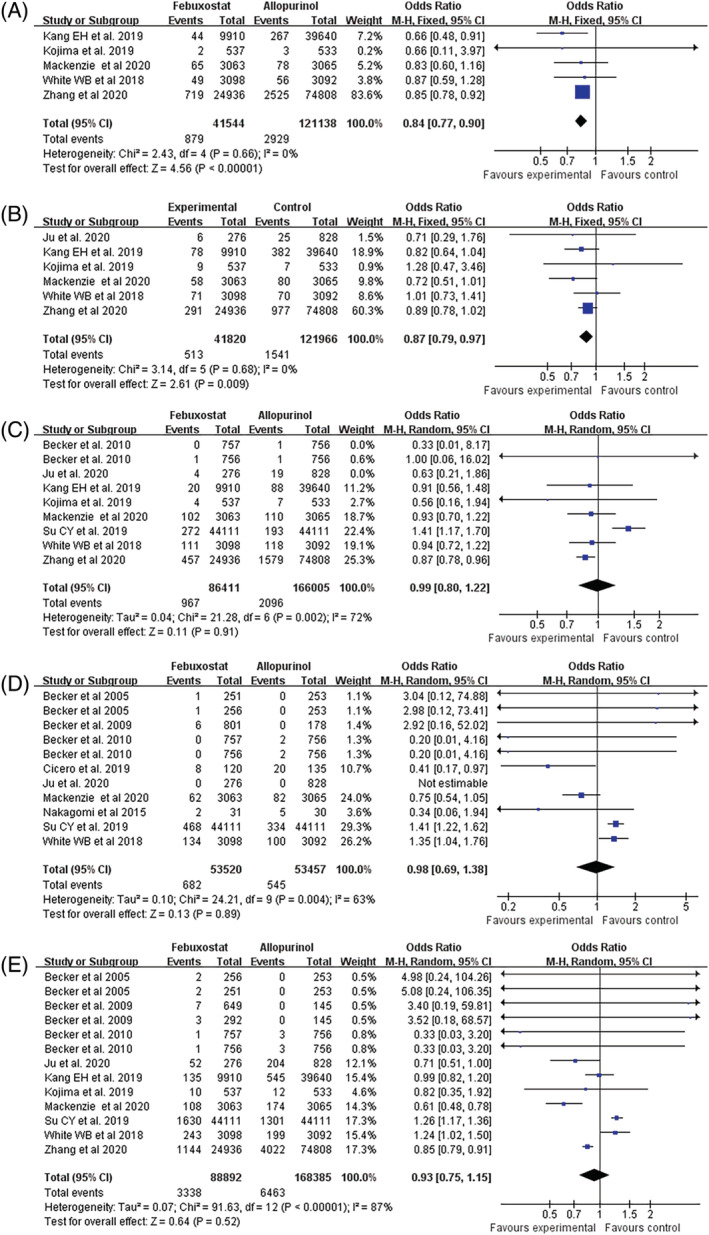
Meta‐analysis of studies that compared the safety in febuxostat therapy and allopurinol treated patients with gout during follow‐up. (A) Urgent Coronary revascularisation; (B) Nonfatal stroke; (C) Nonfatal myocardial infarction; (D) Cardiovascular death; and (E) Death from any cause

Compared with allopurinol treatment group, the febuxostat group had a better safety outcome, which was the composite of urgent coronary revascularization (OR: 0.84, 95% CI: 0.77–0.90, *p* < .0001 Figure 2(A)) and stroke (Figure 2(B)) (OR: 0.87, 95% CI: 0.79–0.97, *p* = .009). However, that difference was not found in nonfatal myocardial infarction (Figure 2(C)) (OR: 0.98 95% CI: 0.80–1.22, *p* = .91), cardiovascular related mortality (Figure 2(D)) (OR: 0.98, 95% CI: 0.69–1.38, *p* = .89) and all‐cause mortality (Figure 2(E)) (OR: 0.93, 95% CI: 0.75–1.15, *p* = .52).

Begg's funnel plot indicated that there are no strong evidences of publication selection bias.

### 3.3 Results of subgroup analyses

To clarify the heterogeneity, subgroup analyses were performed to investigate the source of heterogeneity (Table 2). Compared with allopurinol treatment group, subgroup analyses according to age, population and study design showed that the febuxostat treatment could significantly reduce the occurrence of stroke in age ≥ 65 years group (OR: 0.88, 95% CI: 0.79–0.99, *p* = .03), white race (≥70%) group (OR: 0.88, 95% CI: 0.79–0.99, *p* = .04) and cohort study group (OR: 0.87, 95% CI: 0.78–0.98, *p* = .04). Subgroup analyses according to population showed that the febuxostat treatment could significantly reduce the incidence of nonfatal myocardial infarction in white race participants (OR: 0.87, 95% CI: 0.79–0.96, *p* = .007). No significant differences in cardiovascular related mortality and all‐cause mortality were observed across any subgroup.

**TABLE 2:** Subgroup and sensitivity analyses of adverse events stratified by previously defined study characteristics

Variables	Nonfatal myocardial infarction			Stroke			Cardiovascular related mortality			All‐cause mortality		
Subgroup analysis	No. of trials	OR (95% CI)	*p* for heterogeneity	No. of trials	OR (95% CI)	*p* for heterogeneity	No. of trials	OR (95% CI)	*p* for heterogeneity	No. of trials	OR (95% CI)	*p* for heterogeneity
Age												
<65 years	3	0.89 (0.56, 1.43)	.83	1	0.82 (0.64, 1.04)	Heterogeneity: Not applicable	5	0.99 (0.25, 3.89)	.46	7	1.00 (0.83, 1.20)	.49
≥65 years	6	0.98 (0.77, 1.24)	.0006	5	0.88 (0.79, 0.99)	.59	6	0.97 (0.66, 1.40)	.0004	6	0.91 (0.70, 1.16)	<.0001
Study design												
RCT	5	0.92 (0.76, 1.11)	.91	3	0.88 (0.69, 1.10)	.28	8	0.95 (0.59, 1.52)	.08	9	0.93 (0.57, 1.54)	.001
Cohort study	4	1.00 (0.70, 1.44)	.0001	3	0.87 (0.78, 0.98)	.74	3	0.82 (0.25, 2.71)	.006	4	0.95 (0.73, 1.24)	<.0001
Population												
White Race (≥50%)	4	0.87 (0.79, 0.96)	.88	3	0.88 (0.79, 0.99)	.36	8	0.86 (0.52, 1.41)	.03	9	0.89 (0.66, 1.20)	.0007
Asian	5	1.01 (0.74, 1.39)	.03	3	0.83 (0.66, 1.04)	.65	3	0.92 (0.26, 3.25)	.11	4	0.99 (0.75, 1.29)	.001

## 4 DISCUSSION

The present study suggests compared with allopurinol, the use of febuxostat results in significantly decreased risks of urgent coronary revascularization and stroke. Initiation of febuxostat did not increase the risk of nonfatal myocardial infarction, the cardiovascular related mortality and all‐cause mortality. Subgroup analyses according to age, population and study design showed that the febuxostat treatment could significantly reduce the occurrence of stroke in patients with age ≥65 years and white race (≥70%).

Febuxostat is a urate‐lowering drug that was approved for the management of gout by the EMA, the US FDA and the China FDA (CFDA). The CARES reported an increased risk of death in patients in the febuxostat group, prompting the FDA to change febuxostat's approval status to be adopted only as a second‐line urate‐lowering drug. By contrast, several recent clinical trials do not support the finding of an increased all‐cause mortality and cardiovascular risk of febuxostat.^10^, ^14^, ^24^, ^26^ Our meta‐analysis found that febuxostat users did not have a significantly different risk of cardiovascular events or all‐cause mortality compared with allopurinol users.

Older individuals have higher rates of major cardiovascular events, coexistence of multiple diseases, accompanied by multiple syndromes, multiple medications, and natural decline in body function. Gout currently plagues many old patients. Febuxostat has minimal effects on other enzymes involved in purine and pyrimidine metabolism and is metabolized mainly by glucuronide formation and oxidation in the liver. Febuxostat has been shown to be safe according to the available clinical data and can be used in treating patients with allopurinol hypersensitivity and renal insufficiency.^27^ Since the application of febuxostat to the clinic, there have been many meta‐analysis on the effectiveness and safety of febuxostat compared with allopurinol in the treatment of patients with gout.^28^, ^29^, ^30^, ^31^, ^32^ Cardiovascular events were important concern associated with febuxostat treatment. However, the meta‐analysis of cardiovascular safety of febuxostat compared with allopurinol in the elderly was not reported. Several clinical trials were conducted to investigate the cardiovascular safety of febuxostat compared with allopurinol in patients with gout with known cardiovascular comorbidities, but the results have been conflicting. Some studies showed that the incidence of major adverse cardiovascular events in febuxostat group was numerically higher than allopurinol group, although it was not statistically significantly.^3^, ^4^, ^33^ Some studies found that initiation of febuxostat compared with allopurinol was not associated with a change in risk of cardiovascular events.^14^ The subgroup analysis according to age of our meta‐analysis adds new evidence regarding the safety of febuxostat in older patients.

Although we believe that this meta‐analysis provides useful information, the finding must be interpreted with caution because there are several limitations of this study. First, most of the enrolled allopurinol and febuxostat users with cardiovascular comorbidities or cerebrovascular disease. The basis of inclusion criteria of these studies resulted in different baseline comorbidities. Second, the treatment period and follow‐up time of each study are different, which may cause bias. A longer follow‐up regarding cardiovascular safety should be considered. Third, As the clinical trials included an overwhelming majority of men, febuxostat tolerance remains little explored in women. A careful monitoring of long‐term effects and cardiovascular safety of febuxostat should be considered in female patients.

In conclusion, our meta‐analysis suggested that febuxostat users did not significantly increase the risk of cardiovascular events or all‐cause mortality compared with allopurinol users. However, more high‐quality, double‐blinded, large, randomized studies are needed to elucidate this issue.

## CONFLICT OF INTEREST

The authors declares no conflicts of interest.

## Data Availability

This is an open access article under the terms of the Creative Commons Attribution License, which permits use, distribution and reproduction in any medium, provided the original work is properly cited.
